# The Power of Discourse: Associations between Trainers’ Speech and the Responses of Socialized Wolves and Dogs to Training

**DOI:** 10.3390/ani13061071

**Published:** 2023-03-16

**Authors:** Melissa Gabriela Bravo Fonseca, Heron Oliveira Hilário, Kurt Kotrschal, Friederike Range, Zsófia Virányi, Marina Henriques Lage Duarte, Laryssa Cristina Gomes Pereira, Angélica da Silva Vasconcellos

**Affiliations:** 1 Program of Post-Graduation in Vertebrate Biology, Pontifical Catholic University of Minas Gerais, Belo Horizonte 30535-901, Minas Gerais, Brazil; 2 Laboratory of Conservation Genetics, Pontifical Catholic University of Minas Gerais, Belo Horizonte 30535-901, Minas Gerais, Brazil; 3 Department of Behavioural and Cognitive Biology, University of Vienna, 1010 Vienna, Austria; 4 Domestication Lab, Konrad Lorenz Institute of Ethology, University of Veterinary Medicine Vienna, Savoyenstraße 1a, 1160 Vienna, Austria; 5 Messerli Research Institute—University of Veterinary Medicine Vienna, Medical University of Vienna, University of Vienna, 1210 Vienna, Austria; 6 Bioacoustics Laboratory, Museum of Natural Sciences, Pontifical Catholic University of Minas Gerais, Belo Horizonte 30535-901, Minas Gerais, Brazil

**Keywords:** canids, human-animal interactions, welfare, behavior, acoustics analysis

## Abstract

**Simple Summary:**

In a previous study, we found that Positive Reinforcement Training promoted relaxation in wolves and dogs. Here, we investigate aspects of the trainers’ voices possibly involved in this effect. Dogs’ great interest in high-pitched, intense speech has already been reported, but whether and how wolves respond similarly/differently to voice characteristics has never been studied. We analyzed 270 training sessions with nine mixed-breed dogs and nine wolves. We grouped human speech into three categories: nice, neutral, and reprehensive, and analyzed how their duration and their acoustic characteristics within training sessions were associated with animals’ behavior and physiology. The longer the duration of nice speech during a session, the more often tail wagging was observed in both subspecies, while the opposite was found for reprehensive speech. The duration of reprehensive speech was also associated with a decrease in correct responses in dogs and with retreating in wolves, while a longer use of nice speech was associated with animals being next to the trainer for longer within a session. Sessions with a higher average pitch was more often associated to changes in dog behavior, while wolf behavior changes were more often associated to low intonations. Our results suggest that a friendly voice during training supports performance and positive emotional responses in wolves and dogs. The different response towards the pitch between the subspecies may be related to the domestication process, which selected, in dogs, characteristics that facilitate interaction with humans.

**Abstract:**

In a previous study, we found that Positive Reinforcement Training reduced cortisol of wolves and dogs; however, this effect varied across trainer–animal dyads. Here we investigate whether and how the trainers’ use of speech may contribute to this effect. Dogs’ great interest in high-pitched, intense speech (also known as Dog Directed Speech) has already been reported, but whether and how wolves respond similarly/differently to voice characteristics has never been studied before. We analyzed 270 training sessions, conducted by five trainers, with nine mixed-breed dogs and nine wolves, all human-socialized. Through Generalized Linear Mixed Models, we analyzed the effects of (a) three speech categories (nice, neutral, reprehensive) and laugh; and (b) acoustic characteristics of trainers’ voices on animals’ responses (correct responses, latency, orientation, time at less than 1 m, non-training behaviors, tail position/movements, cortisol variation). In both subspecies, tail wagging occurred more often in sessions with longer durations of nice speech, and less often in sessions with reprehensive speech. For dogs, the duration of reprehensive speech within a session was also negatively related to correct responses. For wolves, retreat time was associated with more reprehensive speech, whereas duration of nice speech was positively associated with time spent within one meter from the trainer. In addition, most dog behavioral responses were associated with higher average intonations within sessions, while wolf responses were correlated with lower intonations within sessions. We did not find any effects of the variables considered on cortisol variation. Our study highlights the relevance of voice tone and speech in a training context on animals’ performances and emotional reactions.

## 1. Introduction

Because humans are extremely communicative, any species that cohabits with us was likely selected to take advantage of this characteristic [1]. Dogs (*Canis familiaris*) are a good model for studying interspecific communication because they can develop and use a flexible signaling system when dealing with humans [2]. Zimen [3] showed that this capacity is derived from wolves. Both subspecies use visual and acoustic signals; however, many canine visual signals involve tail and/or ear movements, which have no counterpart in humans, making the understanding and use of such signals more challenging for us. Likewise, human visual cues often include hand movements for which there is no canine parallel. With regard to acoustic signals, some can result in a lasting communicative pattern based on repeated interactions between sender and receiver [4] and, in the case of dogs, this ability was thought to have been intensified through domestication [5]. Studies have pointed to a synergy between phylogeny and ontogeny in the development of dog abilities, including interspecific communication [6].

Considering that humans are a very vocal species, it is plausible that the communicative approach towards dogs is spontaneously based on such a channel. In Western cultures, it is common for humans to adopt a special type of speech when they “talk” to pets [7], the pet-directed speech (PDS). It shares some of the acoustic characteristics of infant-directed speech (IDS), including an increase in pitch and vowel articulation, in an exaggerated and affected manner, as well as the decrease in the rhythm of words compared to adult-directed speech (ADS). PDS and IDS may show similarities because both dogs and babies are non-verbal listeners, and the affective bond between owners and dogs are known to reflect the human parent–baby bond [8]. In addition, both owners and dogs have been shown to experience oxytocin secretions after a brief period of petting [9], and a study highlighted brain activation of connected areas when mothers viewed images of both their child and their dog [10]. Furthermore, it has been shown that the acoustic characteristics of PDS attract the attention of dogs significantly more than the ADS [8].

Ben-Aderet and colleagues [11] were the first to investigate both the production of speech used specifically for dogs (Dog Directed Speech, DDS), and the behavioral responses of puppies, adult, and old dogs to DDS. They found that although humans produce DDS for animals of different age groups, the preference of dogs for this kind of speech decreases with age: puppies showed greater behavioral responses to DDS than to ADS, while adult and old dogs showed no preference for either type of speech. For the authors, targeting DDS to adult and old dogs may simply constitute a “spontaneous attempt to facilitate interactions with non-verbal listeners”. This interpretation may be related to the “hyperphony” hypothesis [12], according to which broadcasters use optimized speech patterns to improve speech intelligibility with animals, which are expected to be more sensitive to this special modulation of the voice tone.

However, alternative explanations for the lack of effect of DDS on adult dogs still deserve investigation. Ben-Aderet and colleagues [11] suggested that adult dogs may need additional cues (e.g., gestures) to respond to unfamiliar speakers. In their initial study, DDS and ADS were recorded, and only their playbacks were used in the experiments. Therefore, adult dogs did not have the opportunity for receiving extra cues. This condition may have affected communication by influencing sound reception, and precluding dogs from having social interaction; therefore, they may have not seen any social benefit of reacting preferably to any kind of speech. In contrast, puppies, having little experience in terms of environment and social interactions, may lack focus on this aspect, responding to DDS even in the absence of a physical experimenter.

Taking this matter as a starting point, Benjamin & Slocombe [13] carried out an experiment to investigate the possible effects of DDS and ADS on the levels of attention and affiliation of adult dogs. The authors also investigated whether possible behavioral preferences were modulated by prosody and/or content, under conditions ecologically more relevant compared to the study by Ben-Aderet and colleagues [11]. Adult dogs attended more and sought more proximity to an experimenter whose speech had dog-relevant content (e.g., good boy/girl!) and was spoken with elevated pitch and exaggerated prosody than to the experimenter whose speech lacked any of these characteristics. This finding suggested that DDS might fulfill a dual function: improving attention and social connection. This last aspect is in line with the current understanding reached by research with children, which suggests that not only is IDS crucial for the development of meaningful social relationships with caregivers [14], but that it is useful for facilitating language acquisition [15]. Another study has corroborated the results of Benjamin & Slocombe [13] by pointing out that dogs have shown preferences towards a target object associated with DDS in the DDS versus ADS condition [16]. Despite the existence of studies investigating the perception and processing of human vocalizations by dogs, little is known about how their ancestors, the wolves, respond to these stimuli.

In line with the fact that DDS is often characterized by high pitch [7], Ben-Aderet and colleagues [11] also found that dogs reacted more to high pitch than low pitch. Interestingly, however, pitch did not seem to determine their ability to differentiate between DDS and ADS nor did it explain preferences for DDS [16], questioning the importance of single acoustic parameters.

We here focus on communication between humans and animals during Positive Reinforcement Training (PRT), a type of Operant Conditioning technique, which was shown to promote improvements in animal welfare, possibly through the association between the animals’ behavior and its pleasant consequences [17,18]. In essence, PRT may benefit animals by providing positive feedback and, eventually, by promoting opportunities for the animal to exert some control over their environment [19]. However, the beneficial effects recorded with PRT might also be promoted by the opportunity to interact with a familiar person with whom the animal may have developed a social bond [20].

Vasconcellos and colleagues [18] investigated the effects of regular interactions of equally raised and kept timber wolves and mixed-breed dogs with familiar humans during PRT sessions. In addition to their training performance, the animals’ behavioral and physiological responses (salivary cortisol variations) were evaluated. Apart from a fine training performance, a reduction in salivary cortisol concentrations was recorded in both subspecies, as well as low rates of non-training-related behaviors (NTBs) [21]. Interestingly, up to 22.8% of the variation of the animals’ responses were due to trainer identity. These results showed, for the first time, that not only dogs but also wolves may benefit from PRT.

Considering that the way humans communicate/interact with dogs has been shown to affect the owner-dog relationship, animal behavior, and possibly animal welfare [22], in the current study, we explored how animals’ responses might differ in accordance to trainer communication style within a training session. We reasoned that one important point might be the use of voice and thus set out to evaluate the effects of the duration and frequency (number of occurrences) of types of speech used within a training session, and the average acoustic characteristics of trainers’ voices during sessions on animals’ responses. We focused on two hypotheses: (1) the communication style (duration/frequency of nice, neutral or reprehensive speech) and specific acoustic parameters (such as pitch) that characterized the different speech types are associated with different behavioral and physiological responses of dogs and wolves during training; (2) the domestication process affected the perception and responses of animals to the communication style of humans and acoustic sound parameters. From these hypotheses, we developed two predictions: (1) the duration/frequency of “nice” speech and/or high pitch will be associated with behaviors indicative of affability and interest in training, such as increased duration of tail wagging, attention and performance in the animals, and greater proximity of the dyad, whereas (2) the duration/frequency of “reprehensive” speech will be associated with an increase in behaviors unrelated to training, greater distance in the dyad, and duration of the tail being retracted for both subspecies.

## 2. Materials and Methods

### 2.1. Ethics Statement

All study animals were kept at the Wolf Science Center (www.wolfscience.at: license n°: AT00012014). The CITES (www.cites.org) import permits for the animals are 2008: Zoo Herberstein, Austria: AT08-B-0998, AT08-B-0996, AT08-B-0997; 2009: Triple D Farm, USA: AT09-E-0018; 2012: Minnesota Wildlife Connection, USA: 12AT330200INEGCJ93. The animals were housed in accordance with the Austrian Federal Act on the Protection of Animals (Animal Protection Act—TSchG, BGBl. I Nr. 118/2004). Hence, in accordance with the Austrian Animal Experiments Act (BGBl. I Nr. 114/2012, Tierversuchsgesetz 2012—TVG 2012), no ethical approval was officially required, but we still obtained one from the University of São Paulo, Brazil (Committee of Ethics for Animal Research from the Institute of Psychology, University of São Paulo, Brazil, approval number 016.2009).

### 2.2. Subjects

Eighteen animals were studied: nine timber wolves (mean age = 15 months ± 2.04) and nine dogs (mean age = 21 months ± 3.37), all born in captivity, raised and kept following the same protocol [23] at the Wolf Science Center, an institution located in the Game Park Ernstbrunn, Austria. Table 1 shows the animals’ names, sexes, and ages at the onset of the study.

All study animals were hand-raised and maintained in close contact with the five participating trainers during the first 20 weeks of life. From the third week onwards, sporadic contact with conspecifics (including adult individuals, wolves in the case of wolf pups and dogs in the case of dog pups) were provided, while at this age we also started with more formal PRT interactions.

### 2.3. Saliva Collection

Prior to the beginning of the study, dogs and wolves were trained with the use of PRT techniques to allow saliva collection. Saliva was used for the physiological assessment of stress via measuring salivary cortisol. The collection procedure included the introduction of two surgical hydrocellulose sponges (Sorbette, by Salivette^®^) in the animal’s cheek pouch for sufficient time to get these soaked. Immediately after saliva collection, the sponges were transferred to a plastic tube (Sarstedt^®^) and stored at −20 °C until analysis by enzyme immunoassay [18]. During the sessions of familiarization with the collection procedures, as well as during saliva collection and the training sessions, the animals were rewarded exclusively with pieces of Gouda cheese, to control for the influence of protein in the saliva samples [24]. Saliva collection was performed 2–4 min before, and 15 min after the end of each training session.

### 2.4. Procedures

Each study animal participated in 15 training sessions of 5 min each (3 sessions with each of the 5 trainers), totaling 270 sessions, 135 with dogs and 135 with wolves. The training sessions were run between May 2010 and March 2011 and were filmed with a video camera. These were conducted with the animals isolated from the pack, between 8:30 a.m. and 5:00 p.m., in a training room (63.6 m^2^) located close to their enclosure. The training room did not allow visual contact with other animals or humans and was almost empty except for a raised platform on one side. No animal participated in more than one training session per day. Each trainer, however, worked with four to nine animals on each training day, in a randomized order.

All wolves and dogs were already familiar with the cues used during the training sessions (sitting, laying down, turning around, walking around the trainer, giving a paw, allowing the placement of a muzzle, allowing the placement of a harness, rolling, standing, staying, and looking into the trainer’s eyes). The vocal interactions during the sessions occurred in a naturalistic context. Trainers were instructed to remain standing, in a relaxed posture, emitting cues in random order and rewarding the animals when the cues were correctly followed. Animals’ responses were rewarded with cheese in a continuous reinforcement regime. The study was developed to be minimally invasive, in such a way that every animal participated voluntarily in the training sessions, being invited by name to enter the test room. In case of reluctance or discomfort with the procedure, the animal would be reintroduced to the group, where it remained until it was ready to make part of the session.

### 2.5. Animals’ Behaviors and the Types of Speech

**Figure 1:**
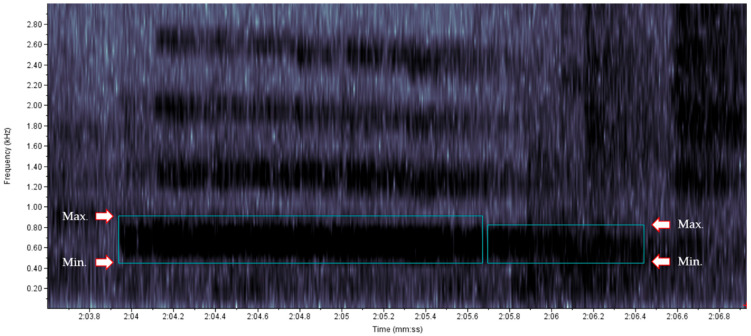
Illustrative spectrogram of a trainer’s speech. Blue boxes represent the marking of the fundamental frequency (F0) of two distinct speech events. Arrows indicate the maximum (max.) and minimum (min.) frequencies of these speeches. X axis: time interval (200 ms); y axis: frequency in kHz.

We evaluated all trainer vocalizations: speeches (phrases uttered by the trainers during the interactions), animal names, and laughs produced by the trainers during the 270 five-minute training sessions, excluding the commands used for training. The commands were not analyzed because their pronunciation was standardized, always in a neutral tone of voice. The start and end of each vocalization were selected both acoustically (by listening to the records), and visually, with the use of spectrograms (Figure 1).

Table 2 contains the description of seven types of trainer vocalization recorded in the videos from the sessions, whose duration or frequency of use was summed up for each training session and considered here as explanatory variables: nice, neutral and reprehensive speeches and names; and laugh. As response variables, we used the same ones evaluated by Vasconcellos et al. [18]: correct responses, latency, visual orientation to trainer, time at less than 1 m from trainer, Non-Training Behaviors (NTB; representing counter-productive behaviors, regarding training), and cortisol variation, in addition to the animals’ tail position/movements (Table 3).

All behavioral parameters considered here were coded from the videos by only one person (author M.G.B.F.—Melissa Gabriela Bravo Fonseca) through Focal Sampling, with Continuous Recording of behaviors, using the Solomon Coder program (Beta version 19.08.02, 2019, by András Péter). To obtain a measure of reliability of the behavior coding, 20% of all videos were re-coded (M.G.B.F.), and the scores compared with those of the first viewing through a Spearman rank correlation; the results indicated a good agreement, with correlation coefficients ranging from 0.86 to 0.99. The variables nice names, neutral names, reprehensive names, correct responses and repetitions were evaluated in terms of frequency (number of occurrences); all other variables were analyzed in terms of duration (seconds).

In order to contribute to the understanding about the possible effects of different types of speech on the animals’ responses, additional exploratory analyses were performed: a comparison among acoustic parameters of the three types of speech, and an analysis of the effects of these separate parameters on the animals’ responses.

### 2.6. Acoustic Comparison among Types of Speech

To investigate acoustic differences between the three types of speech classified in the first stage of the study, we defined six acoustic variables (Table 4). Although all the speeches present all the studied acoustic components (minimum frequency, maximum frequency, peak frequency, average power), we separated the speeches into these components for analysis.

### 2.7. Animals’ Behaviors and Voice Acoustic Characteristics

We extracted audios from the videos recorded during all the 270 training sessions with the Any Video Converter program, in WAV format. The recordings were analyzed using the Raven Pro 1.5 software (Cornell Laboratory of Ornithology—Cornell University), through the analysis of spectrograms.

The fundamental frequency (F0) of the first harmonic of each speech was selected in the spectrogram (Figure 1), and the following parameters were considered for the analysis of F0: (a) Visualization: spectrogram 1; (b) Channel: 1; (c) Brightness: 50%; (d) Contrast: 50%; (e) Size of the spectrogram viewing window: 512; (f) Time interval (“x” axis): 200 ms; and (g) Frequency in kHz (“y” axis): 0 to 2.80. Separate spectrograms for each of the three types of speech analyzed in our study (nice, neutral, and reprehensive) can be seen in the Supplementary Material (Figure S1). For this analysis, the category laugh was considered as a positive non-verbal emotional vocalization [25], and therefore was included in the category “nice”.

For each training session, the averages of the six acoustic variables used to analyze the vocalizations emitted by the trainers (Table 4) constituted the explanatory variables for this stage of the analysis. As response variables in these analyses, we used the same ones described above: correct responses, latency, visual orientation to trainer, time at less than 1 m, Non-Training Behaviors (NTB), and cortisol variation, in addition to the animals’ tail position/movements (Table 3).

### 2.8. Statistical Analysis

The statistical analyses were performed by Generalized Linear Mixed Models (GLMMs), with a Poisson distribution, adjusted for repeated measures using the R software. By adjusting for repeated measures, we could use data from all sessions (3 sessions of each animal with each of the 5 trainers), controlling for trainer and animal repeatability (random factor), summing up 270 training sessions. We used “lme4” [26], “MASS” [27], “car” [28] and “tidyverse” [29] packages to fit GLMM models in R statistical software, version 4.0.1. All results were analyzed based on statistical significance (α ≤ 0.05). We used the iterative method (i.e., starting with the full model and removing explanatory variables with no effect on the response variable). We built two models: GLMM1 to investigate the responses of animals to the three types of speech, and GLMM2 to analyze the animal’s responses to acoustic characteristics of the trainers’ vocalizations.

In the GLMM1 we evaluated the effects of the explanatory variables subspecies, nice names and speeches, neutral names and speeches, reprehensive names and speeches, and laugh on the response variables correct responses, repetitions, latency, visual orientation to trainer, time at less than 1 m, NTBs, tail position/movements, and cortisol variations. With the GLMM2, we evaluated the effects of subspecies, as well as the acoustic variables of the trainers’ vocalizations (minimum frequency, maximum frequency, average power, delta time, peak frequency, and number of speeches) on the response variables correct responses, repetitions, latency, visual orientation to trainer, time at less than 1 m, NTBs, tail position/movements, and cortisol variations.

In both GLMMs, the first model was always built including all data from dogs and wolves, as well as possible two-way interactions between variables. Tables S1 and S2 present the full (initial) models of GLMM1 and GLMM2, respectively. If any explanatory variable showed interaction with the variable subspecies, separate models were run for each subspecies. Pearson correlations were run between the explanatory and the response variables that did not allow the GLMMs to be run (e.g., due to data distribution).

For the acoustic characterization of the three types of speech (nice, neutral, and reprehensive), means and standard deviations of the variables (minimum frequency, maximum frequency, average power, delta time, and peak frequency), in addition to the total number of speeches, were calculated for each type of speech. Subsequently, repeated measures ANOVA with Tukey’s post hoc (for normal distribution of data) or Friedman’s with Dunn’s post hoc (for non-normal data) were run to check for differences among the types of speech, considering each of the acoustic parameters of sound mentioned above.

## 3. Results

### 3.1. Animals’ Behaviors and the Types of Speech

Table S3 presents the GLMM models with the analyses of the animals’ responses to the types of speech, with both subspecies together. Table 5 and Table 6 present the variables that had effects only for dogs and only for wolves, respectively. The response variables retreating and tail retreated, evaluated by Pearson correlations for the two subspecies, are available in Table 7.

**Figure 2:**
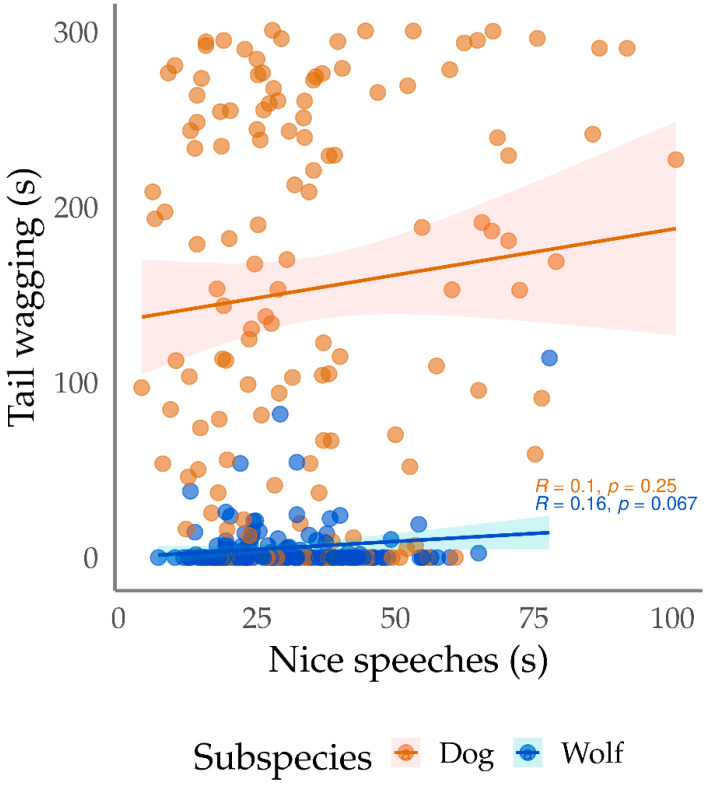
Dispersion of the time that dogs (*Canis familiaris*) and wolves (*Canis lupus*) spent tail wagging in Positive Reinforcement Training sessions, as a function of the duration of “nice speeches” during the sessions. Y axis = tail wagging duration of dogs and wolves (s); X axis = duration of nice speeches (s). Each dot represents an individual training session.

For both dogs and wolves, we identified positive correlations between exploration by the animals and the emission of nice names by the trainer, and also between tail wagging and nice speeches (Figure 2). Regarding tail wagging, while for dogs we identified positive correlations between this variable and neutral speech, for wolves this association was negative. We also recorded negative association between tail wagging and neutral names for dogs, and positive association of tail wagging and neutral names for wolves.

For dogs only, there were direct associations between (a) jumping and neutral speeches, (b) retreating and nice and neutral speeches, and (c) tail retreated and nice names. Negative associations were identified between (d) correct responses and reprehensive speeches and (e) tail wagging and reprehensive speeches.

**Figure 3:**
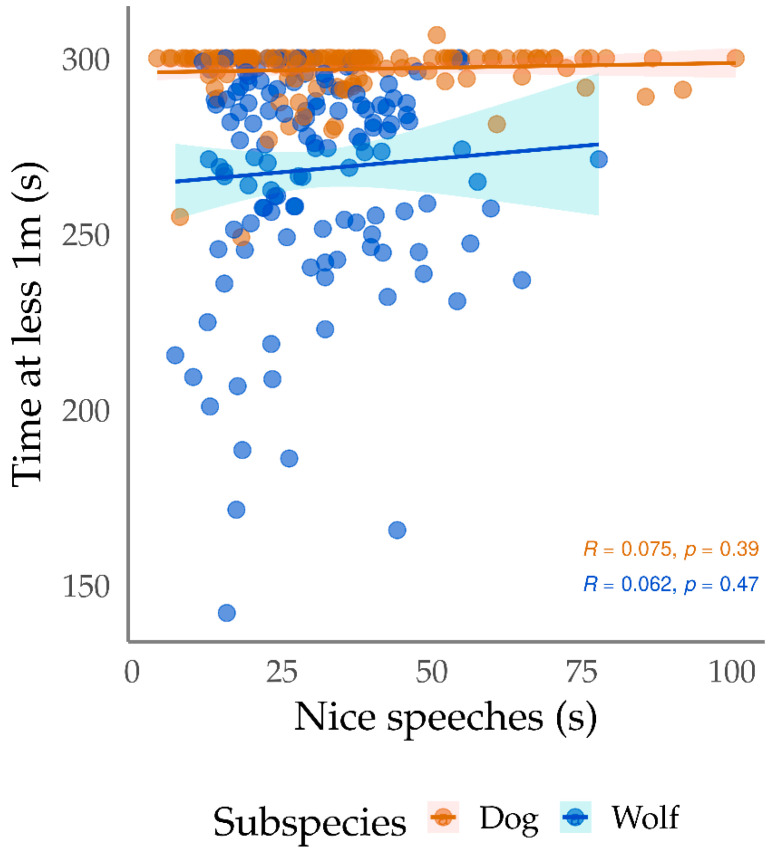
Dispersion of the time that wolves (*Canis lupus*) remained within 1 m from the trainers in Positive Reinforcement Training sessions, as a function of the duration of “nice speeches” during the sessions. Y axis = time at less 1 m (s); X axis = duration of nice speeches (s). Each dot represents an individual training session.

For wolves only, we identified inverse relations between the orientation to trainer and the names emitted in the three types of voice (nice, neutral, and reprehensive). Negative correlations were also observed between the names in the three intonations and the time the wolves spent at less 1 m from the trainer. Still regarding the proximity between the dyad, nice speeches correlated positively with the time the wolves spent within 1 m from the trainers (Figure 3). We also found negative correlations between tail wagging and reprehensive names, and a positive correlation between tail wagging and laughing. Positive correlations were also identified between retreating versus neutral and reprehensive names, as well as between tail retreated and neutral names.

### 3.2. Acoustic Comparison among the Types of Speech

The acoustic comparison among of the three types of speech (nice, neutral, and reprehensive) demonstrated measurable differences in acoustic characteristics (minimum frequency, maximum frequency, delta time, peak frequency, and number of speeches; Table S4). Nice speech was used more often (10.391 times), being more energetic at higher frequencies, and having higher intensity than the neutral speech, but lower intensity than reprehensive speech. Compared to the other categories, the latter had the most extreme values in terms of duration, intensity, sound amplitude, and frequencies. Although we recorded just 47 reprehensive speeches in the 270 sessions, their duration was longer than that of the other types of speech. The neutral speech presented intermediate characteristics between the other two types of speech, being deeper and more energetic at low frequencies. Figure S2 shows the average acoustic characteristics (minimum frequency, maximum frequency, peak frequency, average power, delta time, and number of speeches) of the three different types of speech (nice, neutral, and reprehensive) for each of the five trainers involved in the project (T1–T5).

### 3.3. Animals’ Behaviors and Voice Acoustic Characteristics

Some of the acoustic characteristics of the trainers’ voices showed interaction with subspecies when analyzed for dogs and wolves together, but the effect was not significant in the analysis separated for dogs and wolves. Table S5 presents the full final model for analysis of the animals’ responses to the acoustic characteristics of the trainers’ voices (dogs and wolves together); Table 8 and Table 9 show the final statistical results of the GLMMs for dogs and wolves, respectively. The response variables retreating and tail retreated were evaluated by separate Pearson correlations for each subspecies (Table 10).

**Figure 4:**
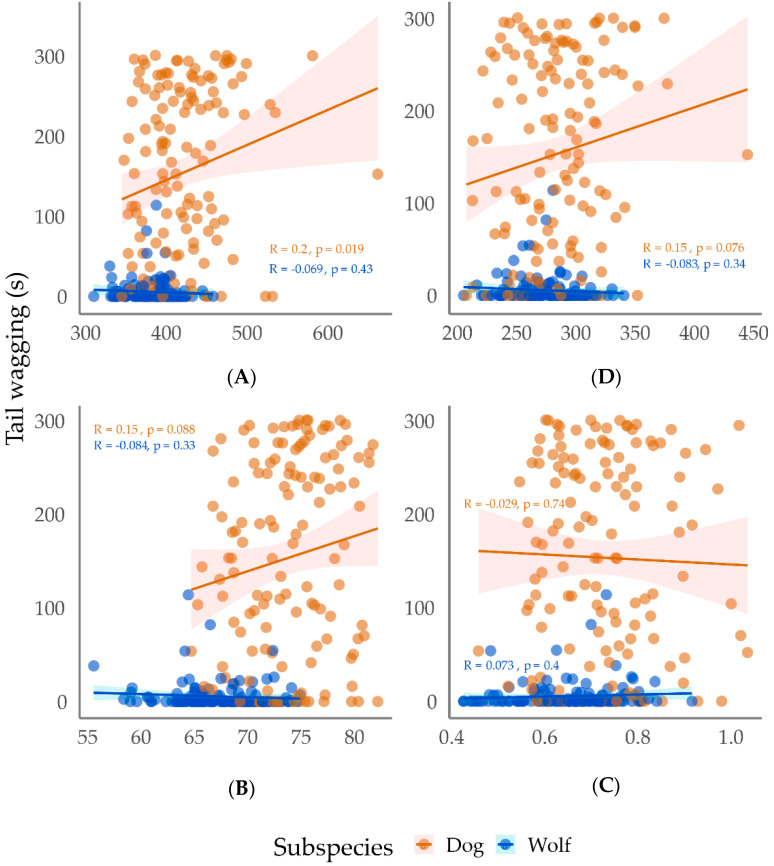
Dispersion of the time dogs (*Canis familiaris*) and wolves (*Canis lupus*) spent tail wagging in Positive Reinforcement Training sessions, as a function of acoustic characteristics of the trainers’ voices. Y axis = tail wagging duration of dogs and wolves (s); X axis = (**A**) maximum frequency (Hz), (**B**) average power (dB), (**C**) delta time (s), and (**D**) peak frequency (Hz). Each dot represents an individual training session.

For both dogs and wolves, we identified positive correlations of tail wagging with maximum frequency and delta time, and an inverse correlation of this variable with average power and peak frequency (Figure 4). In other words, the duration of tail wagging was associated with higher-pitched, longer, and softer speeches (with greater intensity at lower frequencies).

Correct responses, for dogs, were positively related to the measurementes of minimum frequency (higher pitched voices). In this case, this means that in sessions in which the speeches had higher pitch, more correct responses were obtained from the dogs. For wolves, correct responses were inversely associated with minimum frequency and delta time, and directly correlated with peak frequency and the number of speeches, i.e., more correct responses when the session had more speeches, shorter, and in a low pitched tone.

Jumping correlated with maximum frequency and delta time in both subspecies: for dogs jumps were positively associated with higher pitched and longer vocalizations, and, for wolves, this associacion was negative. For wolves, we also identified a direct association between the number of jumps and average power. This means there was more jumping in sessions with more low pitched, intense, and short speeches. Retraction was directly related to the number of speeches (more speeches) in sessions with dogs, and with the mean average power in sessions with wolves (more retraction in the sessions with a greater use of high pitch).

In sessions with wolves, we observed that (a) the visual orientation to trainer was negatively related to minimum frequency, average power and delta time, and directly correlated to peak frequency (greater use of low-pitched, short speeches); (b) the time spent within one meter from the trainer was positively associated to peak frequency, and negatively related to minimum frequency, maximum frequency, and delta time (low-pitched, and short speeches); and (c) tail retracted was directly correlated with maximum frequency (high-pitched speeches).

### 3.4. Cortisol

As described for Vasconcellos and colleagues [18], the cortisol concentrations in the saliva samples taken after the training sessions were lower compared to the samples taken before the sessions in both wolves and dogs (t = −2.864, *p* = 0.004). The mean values for these concentrations were 1023.03 ± 75.99 ng/mL (wolves before training); 820.13 ± 64.03 ng/mL (wolves after training); 2280.87 ± 153.2 ng/mL (dogs before training); and 1851.99 ± 162.9 ng/mL (dogs after training). However, none of the explanatory variables investigated here had a measurable effect on salivary cortisol.

## 4. Discussion

This is a study on the evaluation of the responses of socialized wolves and dogs to human speech during training sessions. Trainers used three distinct types of speech with the animals: nice, neutral, and reprehensive, each with particular acoustic characteristics. Specific features of the prosody and content of the trainers’ speeches correlated to different behaviors from the animals. For both subspecies, the time a trainer used nice speech within a training session was positively correlated to tail wagging, while the duration of reprehensive speech was negatively associated to it. For dogs, the time reprehensive speech was used within a session also negatively related to correct responses. For wolves, retreat occurred more often the more reprehensive speech was used, while they spent greater time within one meter of the trainer in sessions the trainer used nice speech for longer. In addition, dogs tended to respond more to high-pitched voices, while wolves responded more often to low pitch.

### 4.1. Animals’ Behaviors and the Types of Speech

We recorded some similar responses from dogs and wolves to the duration/frequency with which different types of speech were used during the training sessions. The animals wagged their tails more often in sessions in which nice speech was used for longer. This result suggests that friendly interactions between trainer and animal have the potential to improve the emotional conditions of the animals, since tail wagging has already been reported as an expression of a positive emotional disposition (e.g., [30,31,32,33]). The high-pitched tone—one of the aspects that characterizes the nice speech—was also characteristic of DDS-type speeches. DDS has been used to attract the listener’s attention—increasing the recipients’ social responsiveness—and to meet the receiver’s emotional needs, possibly enhancing affiliative communication with the speaker by stimulating affection and emotions with a positive valence [13,34].

Consistent with the animals’ behaviors associated to the nice speech, for both subspecies we recorded that the longer reprehensive speech was used the more negative emotional indicators were observed (reduction in tail-wagging and the time animals spent next to the trainer, and increase in retreat), and the poorer the animals’ performances (e.g., correct responses). Although our training procedures were based on positive reinforcement, it is possible that both dogs and wolves perceived sporadic events of reproachful speech as punishment [35,36]. This perception may have promoted effects on the internal state of the animals [33,36] and a consequent decrease in the rate of correct responses of dogs. Mills and colleagues [37] investigated whether cues with different inflections and emotional content influenced the behavior of dogs. Their results showed that requests pronounced with displeasure or anger were associated to less predictable responses from the animals compared to cues pronounced in neutral or “happy” voice tone. Other evidence indicates that dogs have their behavior influenced by phonetic changes that occur during the emission of verbal cues [38], and that the performance of dogs in solving tasks can be compromised by their emotional dependence on people [39].

For wolves, tail wagging occurred more often when trainers laughed in the training sessions. In the human context, the communication of emotions such as happiness leads each actor to become aware of the other’s euphoric feelings, forging a mutual emotion that acts to cement human social relationships [40]. It is possible that—to some extent—this phenomenon extends to an interspecific scenario. The sections in which moments of relaxation of the trainers—expressed through laughs—were more frequent may have influenced the wolves through contagion. Contagion has been considered adaptive because it allows animals to respond appropriately to different situations, a capacity that favors survival, increases reproductive success [41,42], and has already been described in wolves [43]. Feelings of joy may be communicated through positive facial cues (such as smiling) and vocal cues (laughter) [44,45,46]. Non-verbal interjections—such as laughter, screams, and yelps—contain rich affective information, and can be perceived as the auditory equivalent of facial emotional expressions, which most often accompany these vocal manifestations [47]. Therefore, laughter might have provided a gentler environment, contributing to the relaxation of the wolves [18,36].

We also recorded a positive relationship between the time the trainers used nice speech and the time the wolves spent within one meter from the trainer, suggesting that the use of such a vocal approach may have favored their interest in the training dynamics, bringing the dyad closer together. An alternative interpretation of this association could be that the trainers used softer voices when the animals were attentive and closer to them. However, as preference for DDS has already been demonstrated to influence dog proximity and time spent looking at humans [13,48], we believe the alternative interpretation is unlikely. Although this association has not been demonstrated for wolves so far, the reduction in the time they spent close to the trainers when these used reproachful speech more often during the training sessions is consistent with a greater interest in approaching and interacting with someone using a DDS-type of speech [18,34]. In this study, nice speech was used mainly when gratifying or celebrating a request satisfactorily fulfilled. One can interpret the responses of the animals to the different types of speech as classically conditioned responses (i.e., an association of the nice speech with the food reward). But, in view of the already mentioned and discussed effects of the use of nice and reprehensive speeches, we believe that interpreting the responses of dogs and wolves as purely conditioned behaviors would bring only a limited facet of the situation. Besides, this rationale could not be used to interpret the animals’ responses to reprehensive speech, since this type of speech was not used in situations of incorrect responses to training (and therefore was not connected to withholding a reward). In this sense, we believe that phylogeny and ontogeny interacted in the development of the dogs’ characteristics, favoring interspecific communication such as the type established during the dynamics of PRT, e.g., [6].

Although our analyses have been based on correlations between variables, which do not assess causality, our results point to associations between the environment in a training session (positive: nice speech; negative: reprehensive speech and behaviors of animals) possibly interfering with the animals’ perception of the training context, compliance with requests, attention, and emotional state. It seems that animals interpret cues not only as simple discriminative stimuli, but also as a range of sound signals related to specific contexts including novel physical and social stimuli [49]. This has an important practical effect on the welfare and performance of animals, since variations in the training context might “obscure” the meanings of commands instead of improving understanding, and produce inconsistent feedbacks compared to what was expected [37]. That is, the animals can be influenced by several factors related to verbal communication, such as a reprehensive intonation, for example, leading to responses that are less consistent with the expectations of a training model. These findings recommend the use of a friendly approach even if the animals present responses that are different from the ones requested. This strategy has the potential to create a positive atmosphere, which may favor compliance with commands, and the development—by the animals—of a more positive emotional disposition [36].

Some of our results seem paradoxical at first sight. We found increased jumping and retraction by dogs in sessions when the trainers used more often nice and neutral speeches. This result can be interpreted as an aversive response from dogs to these kinds of speech. This interpretation is possible, but not likely, given that the same types of speech were also associated with a greater duration of tail wagging in dogs and in wolves. Considering that these behaviors can compromise training performance, an alternative—and possibly more sensible—interpretation would be that this result is a consequence of the trainers trying to dissuade the dogs from jumping and retreating, by increasing the duration of the neutral and nice speeches to calm them down, and direct their focus back to the requested cues [36,50,51]. The frequency with which nice names were used in a training session was positively associated to the exhibition of exploration by the animals—a non-training behavior. This association also may have been a delicate and affectionate attempt by the trainers to attract the attention of the animals during moments of dispersal, as observed in dogs in a previous study [36]. The same rationale can be used also to interpret the negative correlation between the frequency of trainers’ calling of wolves’ names in any of the three different intonations (nice, neutral, or reprehensive) with animals’ orientation and time spent within one meter from the trainer. These results suggest that in situations when the wolves were unfocused or not interested in the interactions, the trainers might have increased the frequency of calls to capture the animals’ attention again so that they would return to training [36].

### 4.2. Acoustic Comparison among Types of Speech

This analysis, although explorative in terms of other parameters than pitch, and run to help raise hypotheses for future studies, identified consistent differences between the average acoustic characteristics of the three types of speech recorded. Some of these results corroborate a previous study [52], in which speech constituents with different intonations were investigated. In that study, the “neutral speeches”, in addition to being shorter, had little change in F0, and their outline was smooth and continuous. On the other hand, “angry expressions” were longer and had a higher F0, with more abrupt intervals between one harmonic and another, suggesting these were generated with more emphasis. In addition, the study authors observed that the duration of neutral speeches was shorter than that of angry speeches. Benjamin & Slocombe [13] also argue that DDS-style speeches, compared to ADS-style, have a higher pitch and exaggerated affect, characteristics that were also observed in this study. Therefore, nice speech in our study, to some extent, matched DDS, not only considering its acoustic characteristics, but also regarding dogs’ behaviors associated to it.

Although the nice and reprehensive speeches had some similar characteristics (high-pitched and high-energy at elevated frequencies), we recorded differences between them in other acoustic variables (minimum frequency, maximum frequency, and average power). Different degrees of emotional activation influence tonal and energetic aspects of sound [53,54]. Social stress—such as stress generated during rough interactions, or after unmet expectations—can be responsible for disturbing neurophysiological, behavioral, and emotional patterns of individuals [55]. The differences we found in the acoustic parameters of different speeches may be related to their different valences: a “positive” emotion with physiological arousal versus a “negative” emotion with physiological arousal, respectively [54]. As a result, an increase in the individuals’ respiratory rate and in subglottic pressure during speech increases the relative energy of the upper harmonics and the fundamental frequency. Fundamental frequency (F0—the acoustic element analyzed in this project) is a sound component that usually presents great variation, being indicated for speech analysis regarding the intention and the internal state of the speaker. For example, at high psychophysiological arousal, F0 increases, as well as the amplitude, intensity, variability and rhythm of sound. On the other hand, low excitation patterns are associated with reduced F0, narrow bands, low sound energy, and small variability and slow cadence. In addition, modifications of the F0 height and range have been associated with attentional and affective functions [56]. Increased pitch range (great sound amplitude), as observed in this study in nice speech, is usually related to gaining audience attention and is found in speeches addressed to audiences with limited attention abilities, such as babies or pet dogs [57,58]. It is possible that the lack of attention or objectivity of the animals in certain circumstances has resulted in sporadic events of impatience from the trainers, and therefore in the emission of speeches with different acoustic patterns.

Considering that human voice can be modulated and influenced by several factors intrinsic to the sender or associated with the context, the naturalistic condition in which our study was developed allowed us to unveil the influence of several factors on the animals’ responses to human speech. As the speakers, when modulating the voice, send verbal, non-verbal, and even emotional linguistic information to communicate [55], if our study had been developed based on a predetermined set of sentences and voice intonations, we would possibly have failed to record important nuances associated to the trainers, to the social situation, and to the preferences of the animals, for example [34].

### 4.3. Animals’ Behaviors and Voice Acoustic Characteristics

The acoustic characteristics of the trainers’ voices correlated to different responses from dogs and wolves. In general, dog behavioral changes occurred more often within a session when the trainers talked using high intonations, while most wolf responses were correlated with lower intonations. The study of Ben-Aderet and colleagues [11], the first to investigate the behavioral responses of dogs to DDS, found that puppies prefer DDS to ADS, while adult and old dogs showed no preference for any type of speech—an outcome later extended to adult dogs [13]. Some of the most common pets (i.e., dogs and cats) exhibit neotenous morphological and behavioral characteristics throughout life [59]. The retention of juvenile traits in adulthood (neoteny) is considered a by-product of the domestication process [60,61,62]. Perhaps the tendency of dogs to respond more to high-pitched speeches—a characteristic more intensively exhibited by pups—is associated with neotenic patterns, in which there is a preference for more affectionate vocal interactions. Furthermore, dogs’ preferences for heightened prosodic traits may have an evolutionary explanation. Mammals are known to use acoustic signals to demonstrate motivations, intentions, and emotional states in specific contexts [63,64]. For example, high tonal sounds are associated with affiliative or submissive motivation probably because they imitate the sounds produced by babies (leading to a calming effect over the receiver) [65].

An alternative explanation for the preference of dogs for high-pitched voices is a possible selection of auditory capabilities. Human beings are a very vocal species; we rely on this communicative channel to establish interactions with conspecifics and other social partners, and this extends to pets. Humans not only talk to them, but also teach them to respond to certain acoustic signals [2]. As demonstrated in other studies, some characteristics of DDS (e.g., greater pitch, used in an exaggerated and affected manner, and a slower rhythm compared to ADS) are associated with the establishment and maintenance of social connections between sender and receiver. Dogs’ preference for DDS over ADS [13], and the demonstration that the neural activation in face of positive valence vocalizations in dogs is similar to the activation in humans [1] suggest dogs may have a particular perception of human voice. Such a perception may have improved during domestication, and therefore become more sophisticated compared to wolves.

It is also possible that dogs, due to their great dependence on humans [66], noticing the trainers’ proneness to friendly interactions, showed pro-social behaviors, to strengthen bonds with them [13]. Attachment between a dog and its human attachment figure creates a secure relationship for both [67,68], and can interfere with behavioral patterns associated with loyalty and the desire to please this human [67,68,69]. From an evolutionary perspective, it is likely that dogs that responded to humans emitting high-pitched speeches might have obtained greater fitness than those that did not respond to them. Therefore, this type of interaction might have been essential to the animals during the first stages of domestication, acting in the establishment of a strong bond with humans and thus increasing the chance of the animals obtaining better resources. Hasting [70] explored how human communicative behavior may have influenced the evolutionary process of domestication of dogs by analyzing the use of motherese speech, a DDS-equivalent, also often used by humans when interacting with their dogs. Observing the effectiveness of motherese in human encounters with socialized wolves, the author speculated that dog domestication may have been influenced by human auditory communication patterns. She compared the behavioral reactions of captive wolves, dogs, and hybrids of these two subspecies by using separate auditory stimulus patterns: motherese, normal speeches, and mute (no vocal stimuli), and recorded a strong preference of dogs for motherese, while wolves and wolf-dog hybrids showed no preference for any auditory stimulus over another. Our outcomes corroborate Hasting’s work [70] in regard to dogs’ reactions to high-pitched, intense speech, and points to a different pattern in wolves—a greater interest in low-pitched speeches—likely due to the lack in this subspecies of the domestication effects [71]. These results may be taken as a standpoint for future studies.

### 4.4. Study Limitations

One limitation of this study is that we did not analyze the direct responses of animals towards human speech, but rather how the duration/frequency of the type of speech within 5 min training sessions might have influenced the animals’ behaviors. Differently from other studies e.g., [11,13], we can draw no conclusions on how a certain type of speech directly influences the behavior of the animals. Thus, we only draw conclusions of how the general atmosphere in the training sessions (e.g., the duration of the different types of speech) might have influenced the animal’s reactions.

The responses of the animals in training sessions with more reproachful speech indicated possible impacts of this type of discourse. However, reprehensive speeches were much less frequently used (47 times in 270 sessions) than nice (10.281 times) and neutral (7.500 times) speeches, limiting conclusions. It is possible that characteristics of the trainer, such as preference for certain individuals/subspecies have modulated their use of speech. This type of limitation—common in naturalistic research—can be overcome in future (non-naturalistic) studies by standardizing/equalizing the use of the different speeches. The possible confounding influence of learned responses in the reactions of the animals to training may be minimized by including in the sample naïve animals—i.e., animals not used to training, or by evaluating the animals’ responses to human speech out of the training context. Our sample, although among the average sizes of studies on socialized wolves, e.g., [72,73,74], is not large compared to studies on domesticated species. In order to deal with this constraint, we adjusted our analysis for repeated measures, therefore making it possible to include data from all 270 sessions in the analysis. Finally, it is known that communication usually occurs in a multimodal context. For example, studies have indicated an intermodal ability in dogs related to the integration of visual and auditory emotional cues [75,76]. The focus of our study has been on the association between dogs’ and wolves’ behavior and human speech, not investigating other communicative channels. Future studies should integrate analysis on responses to vocalization with other factors involved in communication, such as gestures, gaze direction, posture, and facial expressions. Such studies have the potential to still improve our understanding of interspecific communication during training.

## 5. Conclusions

The trainers in our study used three distinct types of speech with the animals: nice, neutral, and reprehensive, each with distinct acoustic characteristics. Nice speech was associated with the exhibition by the animals of behaviors indicative of confidence, agreeableness, positive arousal, and proximity between trainer and animal. Reprehensive speech had an opposite effect, having been associated with a decrease in tail wagging, in the rate of correct responses, and with increased retreat. These results reiterate the importance of considering vocal intonation as an important factor in interactions with canids (and possibly other species). Hence, we identified some factors that have the potential for optimizing training conditions, considering both animal performance and the establishment of pleasant interactions between them and their human partners. In addition, our results indicated that most dog responses were associated with high intonations, while wolf responses were correlated with low intonations, a phenomenon possibly associated to the domestication process, which may have favored in dogs the exhibition of neotenic patterns.

## Figures and Tables

**Table 1:** Names, sexes *, and ages (in months, at the moment of data collection) of the dogs and wolves that participated of Positive Reinforcement Training sessions in this study.

Wolves	Age (Months)	Dogs	Age (Months)
Apache (M)	11	Alika (F)	12
Aragorn (M)	24	Kilio (M)	11
Cherokee (M)	11	Layla (F)	24
Geronimo (M)	11	Maisha (M)	11
Kaspar (M)	24	Meru (M)	43
Nanuk (M)	11	Nia (F)	25
Shima (F)	24	Nuru (M)	26
Tatonga (F)	11	Rafiki (M)	12
Yukon (F)	11	Zuri (F)	26

* M = male, F = female.

**Table 2:** Description of seven types of trainer vocalization, obtained for each training session with socialized dogs (*Canis familiaris*) and wolves (*Canis lupus*).

Parameters	Description
Nice names	Trainer calling the animals’ names in a motivating or exalted way. Use of a high tone of voice.
Nice speeches	Trainer speaking to the canids in a motivating or exalted way, usually when “gratifying” or “celebrating” a request satisfactorily fulfilled. Use of a high tone of voice.
Neutral names	Trainer calling the animals’ names with a neutral intonation, similar to that used to request cues during training. Use of words without intense variations in intonation.
Neutral speeches	Trainer speaking to the canids with a neutral intonation, similar to that used to request cues during training. Use of words without intense variations in intonation.
Reprehensive names	Trainer calling the animals’ names with a high, reproachful tone of voice, usually when inappropriate behaviors were exhibited by the animals, such as dispersion, inattention, etc.
Reprehensive speeches	Trainer speaking to the canids with a high, reproachful tone of voice, usually when inappropriate behaviors were exhibited by the animals, such as dispersion, inattention, etc.
Laugh	Trainers’ laughter, produced usually in moments of relaxation, when interacting in a playful manner with the animals or when they did something funny.

**Table 3:** Description of the behaviors of socialized dogs (*Canis familiaris*) and wolves (*Canis lupus*), obtained for each training session.

Parameters	Description
Correct responses	Mean number of cues correctly responded at first request.
Repetitions	Mean number of repetitions of the cue when the animal did not respond to it at first request.
Latency	Average intervals between cue request and the start of the expected response.
Visual orientation to trainer	Mean time the animal spent oriented towards the trainer (the orientation of its head deviating less than 10° from the trainer’s face).
Time at less than 1 m	Mean time the animal spent within 1 m from the trainer.
Non-Training Behaviors (NTBs)	Exploring: animal sniffs the ground or the walls.
	Jumping: animal is either standing on its hind legs or jumping, with its four legs leaving the ground, and touches the trainer with its forelegs.
	Retreating: animal moves away from the trainer, abandoning the interaction.
Cortisol variations	Difference between cortisol concentrations in the salivary samples collected before and after the sessions.
Tail position/movements	Retreated: tail pulled between the legs.
	Wagging: tail moving from side to side.

**Table 4:** Description of the acoustic variables used to characterize the trainers’ vocalizations extracted from videos from training sessions with socialized dogs (*Canis familiaris*) and wolves (*Canis lupus*).

Parameters	Description
Minimum frequency	Measurement, in hertz (Hz), of the lowest frequency of the first harmonic of the speech.
Maximum frequency	Measurement, in Hz, of the highest frequency of the first harmonic of the speech.
Average power	Average value, in decibels (dB), of the power of the sound contained in the first harmonic of the speech.
Delta time	Duration, in seconds (s), of the speech, calculated by the difference between the end time and the begin time.
Peak frequency	Measurement, in Hz, of the frequency with the highest energy present in the first harmonic of the speech.
Number of speeches	Sum of the number of speeches emitted by the trainers per session.

**Table 5:** Final Generalized Linear Mixed Models * run for dogs (*Canis familiaris*), evaluating the effects of the variables nice speeches, nice names, neutral speeches, neutral names, and reprehensive speeches on the response variables correct responses, exploring, jumping, and tail wagging.

Response Variables	Explanatory Variables	Estimate ± sd	z-Value	*p*
Correct responses	(intercept)	3.83 ± 0.03	110.66	0.000
	Reprehensive speeches	−0.95 ± 0.33	−2.80	0.005
Exploring	(intercept)	−0.91 ± 0.38	−2.37	1.75
	Nice names	0.28 ± 0.04	7.50	<0.0001
Jumping	(intercept)	−1.31 ± 0.42	−3.08	0.002
	Neutral speeches	0.04 ± 0.01	3.04	0.002
Tail wagging	(intercept)	4.21 ± 0.34	12.51	<0.0001
	Nice speeches	0.003 ± 0.000	7.53	<0.0001
	Neutral speeches	0.006 ± 0.001	4.37	<0.0001
	Reprehensive speeches	−1.68 ± 0.24	−7.00	<0.0001
	Neutral names	−0.014 ± 0.003	−4.13	<0.0001

* Explanatory variables without effect on the response variables were removed from the models during the model selection process.

**Table 6:** Final Generalized Linear Mixed Models * run for wolves (*Canis lupus*), evaluating the effects of the variable nice speeches, nice names, neutral speeches, neutral names, reprehensive names, and laugh on the response variables visual orientation to trainer, time at less than 1 m, exploring, and tail wagging.

Response Variables	Explanatory Variables	Estimate ± sd	z-Value	*p*
Visual orientation to trainer	(intercept)	5.64 ± 0.33	16.90	<0.0001
	Nice names	−0.021 ± 0.004	−5.51	<0.0001
	Neutral names	−0.016 ± 0.001	−10.44	<0.0001
	Reprehensive names	−0.034 ± 0.009	−3.64	<0.0001
Time at less than 1 m	(intercept)	5.61 ± 0.33	16.82	<0.0001
	Nice speeches	0.002 ± 0.000	5.07	<0.0001
	Nice names	−0.018 ± 0.004	−4.78	<0.0001
	Neutral names	−0.011 ± 0.001	−7.49	<0.0001
	Reprehensive names	−0.019 ± 0.009	−2.23	0.002
Exploring	(intercept)	2.90 ± 0.33	8.68	<0.0001
	Nice names	0.08 ± 0.01	7.37	<0.0001
Tail wagging	(intercept)	0.87 ± 0.35	2.46	0.01
	Nice speeches	0.011 ± 0.002	4.26	<0.0001
	Neutral speeches	−0.020 ± 0.005	−4.12	<0.0001
	Neutral names	0.026 ± 0.01	2.71	0.007
	Reprehensive names	−0.57 ± 0.14	−4.22	<0.0001
	Laugh	0.05 ± 0.02	2.91	0.003

* Explanatory variables without effect on the response variables were removed from the models during the model selection process.

**Table 7:** Pearson correlations run for dogs (*Canis familiaris*) and wolves (*Canis lupus*), considering the effects of the variables nice speeches, nice names, neutral speeches, neutral names, and reprehensive names on the response variables retreating and tail retreated.

Subspecies	Response Variables	Explanatory Variables	Pearson	*p*-Value
Dog	Retreating	Nice speeches	0.23	0.01
		Neutral speeches	0.18	0.04
Wolf	Retreating	Neutral names	0.30	0.28
		Reprehensive names	<0.001	<0.001
Dog	Tail retreated	Nice names	0.22	0.01
Wolf	Tail retreated	Neutral names	0.17	0.05

**Table 8:** Final Generalized Linear Mixed Models * run for dogs (*Canis familiaris*), analyzing the effects of the variables minimum frequency; maximum frequency; delta time; average power; peak frequency on the response variables correct responses; jumping; and tail wagging.

Response Variables	Explanatory Variables	Estimate ± sd	z-Value	*p*
Correct responses	(intercept)	3.61 ± 0.07	12.65	0.00
	Minimum frequency	0.002 ± 0.000	3.78	<0.001
Jumping	(intercept)	−5.17± 0.99	−5.21	<0.001
	Maximum frequency	0.005 ± 0.001	3.32	<0.001
	Delta time	3.13 ± 0.98	3.18	0.001
Tail wagging	(intercept)	4.10 ± 0.40	10.18	<0.001
	Maximum frequency	0.003 ± 0.000	7.67	<0.001
	Average power	−0.01 ± 0.00	−2.94	0.003
	Delta time	0.40 ± 0.07	5.39	<0.001
	Peak frequency	−0.003 ± 0.000	−4.42	<0.001

* Explanatory variables without effect on the response variables were removed from the models during the model selection process.

**Table 9:** Final Generalized Linear Mixed Models * run for wolves (*Canis lupus*), analyzing the effects of the variables minimum frequency; maximum frequency; delta time; average power; peak frequency and number of speeches on the response variables correct responses; visual orientation to trainer; time at less than 1 m; jumping; and tail wagging.

Response Variables	Explanatory Variables	Estimate ± sd	z-Value	*p*
Correct responses	(intercept)	3.17 ± 0.25	12.65	<0.001
	Minimum frequency	−0.004 ± 0.001	−3.70	<0.001
	Delta time	−1.13 ± 0.20	−5.50	<0.001
	Peak frequency	0.005 ± 0.001	4.52	<0.001
	Number of speeches	0.002 ± 0.001	2.27	0.023
Visual orientation to trainer	(intercept)	6.48 ± 0.35	18.33	<0.001
	Minimum frequency	−0.003 ± 0.000	−5.73	<0.001
	Average power	−0.01 ± 0.00	−8.84	<0.001
	Delta time	−0.20 ± 0.06	−3.52	<0.001
	Peak frequency	0.002 ± 0.000	4.43	<0.001
Time at less than 1 m	(intercept)	5.90 ± 0.34	17.13	<0.001
	Minimum frequency	−0.002 ± 0.000	−3.78	<0.001
	Maximum frequency	−0.002 ± 0.000	−6.28	<0.001
	Delta time	−0.15 ± 0.05	−2.81	0.005
	Peak frequency	0.003 ± 0.000	5.30	<0.001
Jumping	(intercept)	0.23 ± 1.70	0.14	0.891
	Maximum frequency	−0.02 ± 0.00	−6.52	<0.001
	Average power	0.15 ± 0.03	4.87	<0.001
	Delta time	−2.30 ± 0.97	−2.38	0.017
Tail wagging	(intercept)	4.15 ± 0.80	5.17	<0.001
	Maximum frequency	0.030 ± 0.003	8.22	<0.001
	Average power	−0.12 ± 0.013	−8.88	<0.001
	Delta time	3.30 ± 0.41	8.09	<0.001
	Peak frequency	−0.03 ± 0.00	−9.29	<0.001

* Explanatory variables without effect on the response variables were removed from the models during the model selection process.

**Table 10:** Pearson correlations run for dogs (*Canis familiaris*) and wolves (*Canis lupus*), considering the effects of the acoustic characteristics of the trainers’ voices (average power, number of speeches, and maximum frequency) on the responses of the animals to training (retreating and tail retreated).

Subspecies	Response Variables	Explanatory Variables	Pearson	*p*-Value
Dog	Retreating	Number of speeches	0.21	0.01
Wolf	Retreating	Average power	0.26	<0.001
Wolf	Tail retreated	Maximum frequency	−0.17	0.05

## Data Availability

Data available on request due to restrictions (e.g., privacy or ethical). The data presented in this study are available on request from the corresponding author. The data are not publicly available due to privacy restrictions.

## Supplementary Materials

The following supporting information can be downloaded at: https://www.mdpi.com/article/10.3390/ani13061071/s1, Figure S1: Illustrative spectrogram of trainer’s speeches. Red boxes represent the marking of the fundamental frequency (F0) of distinct speech events in the three categories studied: nice (A), neuter (B) and reprehensive (C). Arrows indicate the maximum (max.) and minimum (min.) frequencies of these speeches. X axis: time interval of 200 ms; y axis: frequency in kHz; Table S1. Full (initial) Generalized Linear Mixed Models run for dogs (*Canis familiaris*) and wolves (*Canis lupus*), considering the effects of the explanatory variables (subspecies; nice names—NiN; neutral names—NeN; reprehensive names—ReN; nice speeches—NiS; neutral speeches—NeS; reprehensive speeches—ReS; and laugh—L) on the response variables (visual orientation to trainer—vot; exploring—exp; jumping—jump; time at less than 1 m—less; correct responses—correct; and tail wagging—waggp); Table S2. Full (initial) Generalized Linear Mixed Models run for dogs (*Canis familiaris*) and wolves (*Canis lupus*), considering the effects of the explanatory variables (subspecies; minimum frequency (Min_Freq); maximum frequency (Max_Freq); average power (Avg_Pow); delta time (delta_time); peak frequency (Peak_Freq); and number of speeches (num_speeches) on the response variables (visual orientation to trainer—vot; exploring—exp; jumping—jump; time at less than 1 m—less; correct responses—correct; and tail wagging—waggp); Table S3. Final Generalized Linear Mixed Models run for dogs (*Canis familiaris*) and wolves (*Canis lupus*), considering the effects of the explanatory variables (subspecies; nice names—NiN; neutral names—NeN; reprehensive names—ReN; nice speeches—NiS; neutral speeches—NeS; reprehensive speeches—ReS; and laugh—L) on the response variables (visual orientation to trainer; exploring; jumping; time at less than 1 m; correct responses; and tail wagging); Table S4. Results of Dunn and Tukey *post hoc* tests comparing the acoustic parameters (minimum frequency, maximum frequency, delta time, peak frequency, number of speeches, average power) of nice, neutral and reprehensive speeches emitted by trainers in Positive Reinforcement Training sessions with dogs (*Canis familiaris*) and wolves (*Canis lupus*); Table S5. Final Generalized Linear Mixed Models run for dogs (*Canis familiaris*) and wolves (*Canis lupus*), considering the effects of the explanatory variables (subspecies; minimum frequency—MinF; maximum frequency—MaxF; peak frequency—PF; average power—AP; delta time—DT; and number of speeches—NS) on the response variables (visual orientation to trainer; exploring; jumping; time at less than 1 m; correct responses; and tail wagging); Figure S2. Characterization of voice of the five trainers involved in Positive Reinforcement Training sessions with dogs (*Canis familiaris*) and wolves (*Canis lupus*). Left to right, bottom margin: trainers 1, 2, 3, 4, and 5. Acoustic parameters of trainers’ voices: minimum frequency, maximum frequency, peak frequency, average power, delta time, and number of speeches. Number of nice, neutral, and reprehensive speeches: 10.391, 7.500, and 47, respectively.
