# Supplementary material for: The Power of Discourse: Associations between Trainers’ Speech and the Responses of Socialized Wolves and Dogs to Training
Source: Animals (Basel). 2023 Mar 16;13(6):1071. doi: 10.3390/ani13061071 (PMC10044238; doi:10.3390/ani13061071)

Supplementary material

The power of discourse: associations between trainers' speech and behavioral and physiological responses of socialized wolves (*Canis lupus*) and dogs (*Canis familiaris*) to training.

Fonseca, MGB; Hilário, HO; Kotrschal, K; Range, F; Virányi, Z; Duarte, MHL; Pereira, LG; Vasconcellos, AS

Figure S1: Illustrative spectrogram of trainer's speeches. Red boxes represent the marking of the fundamental frequency (F0) of distinct speech events in the three categories studied: nice (A), neuter (B) and reprehensive (C). Arrows indicate the maximum (max.) and minimum (min.) frequencies of these speeches. X axis: time interval of 200 milliseconds; y axis: frequency in kHz.

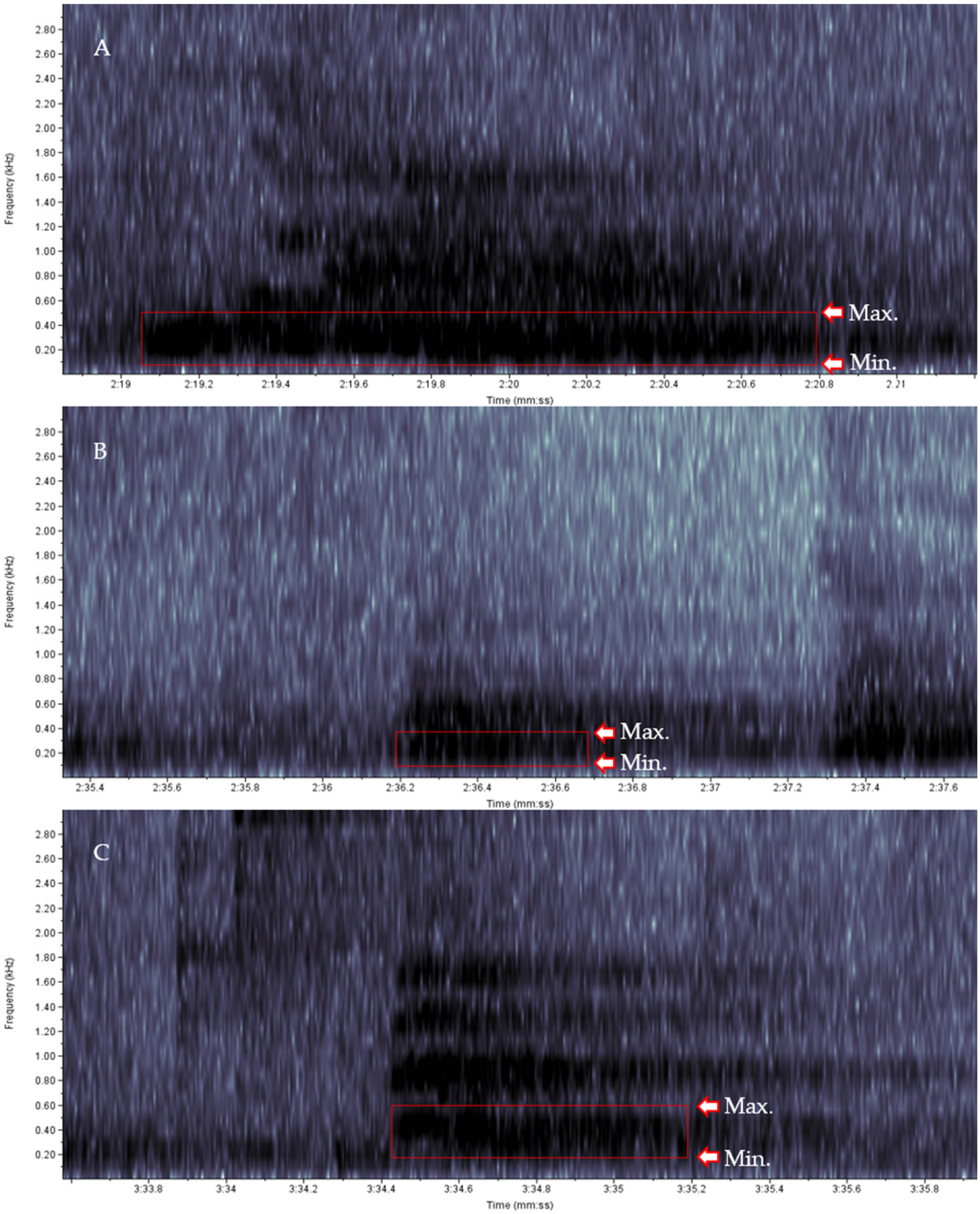

Table S1. Full (initial) Generalized Linear Mixed Models* run for dogs (*Canis familiaris*) and wolves (*Canis lupus*), considering the effects of the explanatory variables (subspecies; nice names – NiN; neutral names – NeN; reprehensive names – ReN; nice speeches – NiS; neutral speeches – NeS; reprehensive speeches – ReS; and laugh – L) on the response variables (visual orientation to trainer – vot; exploring – exp; jumping – jump; time at less than 1 meter – less; correct responses – correct; and tail wagging – waggp).

Model	Intercept	Subspecies**	NiS	NeS	NiN	NeN	ReS	ReN	L	Subspecies: NiS	Subspecies: NeS	Subspecies: NiN	Subspecies: NeN	Subspecies: ReS	Subspecies: L
mod_exp voice_art	5.72E-18	2.80E-13	8.02E-11	5.56E-02	1.18E-06	2.91E-42	7.85E-01	1.41E-01	2.86E-01	9.01E-02	1.10E-02	6.82E-07	6.53E-01	9.83E-01	6.04E-02
mod_correct_voice_art	0.00E+00	2.90E-03	1.82E-01	1.22E-05	2.61E-01	1.48E-01	1.36E-01	8.92E-01	4.20E-02	3.26E-01	1.33E-01	5.20E-01	3.40E-01	5.87E-03	2.55E-02
mod_jump_voice_art	7.37E-01	4.43E-02	1.49E-01	1.20E-02	4.52E-02	6.36E-02	5.34E-07	9.97E-01	1.49E-02	9.19E-01	5.35E-03	8.60E-02	6.71E-02	9.88E-01	9.19E-02
mod_less _voice_art	1.84E-63	8.62E-01	1.27E-06	9.04E-01	3.43E-06	8.98E-14	1.76E-01	2.27E-02	9.98E-01	3.52E-04	8.15E-01	1.19E-03	5.49E-04	6.78E-01	5.90E-01
mod_vot_voice_art	8.99E-63	8.13E-01	1.73E-02	6.59E-02	1.05E-09	3.57E-27	9.82E-02	1.78E-03	2.61E-01	8.55E-02	2.94E-01	3.61E-05	5.23E-07	9.51E-02	2.21E-01
mod_waggp_art_voice	1.42E-02	9.40E-12	2.49E-05	9.54E-05	8.68E-01	5.88E-03	4.91E-01	4.29E-05	3.11E-03	7.37E-03	1.86E-06	3.56E-01	3.46E-04	3.15E-11	7.51E-03

* Model assumptions were tested through graphical evaluation, and correlations between pairs of variables were previously checked to avoid collinearity. No pairs of variables presented correlations equal or greater than 0.7.

** Wolves or dogs; wolves is the reference group.

Table S2. Full (initial) Generalized Linear Mixed Models* run for dogs (*Canis familiaris*) and wolves (*Canis lupus*), considering the effects of the explanatory variables (subspecies; minimum frequency (Min_Freq); maximum frequency (Max_Freq); average power (Avg_Pow); delta time (delta_time); peak frequency (Peak_Freq); and number of speeches (num_speeches) on the response variables (visual orientation to trainer – vot; exploring – exp; jumping – jump; time at less than 1 meter – less; correct responses – correct; and tail wagging – waggp).

Model	Intercept	Subspecies*	all_mean Min_Freq	all_mean Max_Freq	all_mean Avg_Pow	all_mean delta_time	all_mean Peak_Freq	all num_speeches	Subspecies: all_mean Min_Freq	Subspecies: all_ mean Max_Freq	Subspecies: all_mean Avg_Pow	Subspecies: all_mean delta_time	Subspecies: all_mean Peak_Freq	Subspecies: all_num speeches
mod_exp_acoustic	6.88E-05	2.60E-01	1.13E-15	1.16E-04	5.17E-01	3.02E-02	2.12E-10	1.59E-02	4.95E-01	5.79E-02	4.29E-01	4.17E-01	9.74E-01	1.29E-05
mod_correct_acoustic	6.67E-20	1.19E-01	5.98E-04	3.49E-02	6.92E-02	4.66E-07	1.30E-01	8.94E-03	1.07E-05	3.45E-01	7.98E-01	1.36E-04	1.76E-02	6.57E-03
mod_jump_acoustic	2.95E-01	4.58E-01	2.16E-03	1.21E-02	3.34E-02	8.81E-03	2.17E-01	1.49E-01	1.76E-01	1.53E-03	4.02E-02	4.90E-05	1.54E-01	4.67E-01
mod_less_acoustic	1.58E-67	3.13E-01	2.92E-05	2.58E-02	1.77E-03	3.99E-02	3.43E-04	1.50E-01	9.44E-05	2.83E-02	6.18E-02	2.04E-02	3.65E-04	1.69E-01

mod_vot_acoustic	1.22E-73	1.13E-01	4.96E-09	3.83E-01	1.62E-08	1.91E-04	4.92E-04	2.46E-01	3.11E-08	3.23E-01	5.74E-04	4.48E-04	7.16E-04	1.81E-01
mod_waggp_acoustic	4.98E-04	5.41E-01	1.78E-02	2.47E-14	3.14E-09	1.16E-14	1.58E-16	4.32E-01	8.71E-02	1.40E-11	2.70E-07	9.97E-12	5.82E-13	9.95E-02

* Model assumptions were tested through graphical evaluation, and correlations between pairs of variables were previously checked to avoid collinearity. No pairs of variables presented correlations equal or greater than 0.7.

** Wolves or dogs; wolves is the reference group.

Table S3. Final Generalized Linear Mixed Models* run for dogs (*Canis familiaris*) and wolves (*Canis lupus*), considering the effects of the explanatory variables (subspecies; nice names – NiN; neutral names – NeN; reprehensive names – ReN; nice speeches – NiS; neutral speeches – NeS; reprehensive speeches – ReS and laugh – L) on the response variables (visual orientation to trainer; exploring; jumping; time at less than 1 meter; correct responses and tail wagging).

Response variables	Explanatory variables	Estimate ± sd	z-value	*p*
Visual orientation to trainer	(intercept)	5.58 ± 0.746	7.48	<0.001
	Subspecies**	0.06 ± 0.472	0.12	0.903
	NiN	-0.04 ± 0.009	-4.87	<0.001
	NeN	-0.03 ± 0.004	-7.65	<0.001
	ReN	-0.03 ± 0.009	-3.64	<0.001
	Subspecies X NiN	0.02 ± 0.005	3.84	<0.001
	Subspecies X NeN	0.01 ± 0.003	4.81	<0.001
Exploring	(intercept)	6.22 ± 0.771	8.07	<0.001
	Subspecies	-3.65 ± 0.508	-7.18	<0.001
	NiN	-0.19 ± 0.043	-4.37	<0.001
	NeN	0.06 ± 0.004	14.04	<0.001
	Subspecies X NiN	0.22 ± 0.039	5.64	<0.001
Jumping	(intercept)	1.67 ± 0.882	1.89	0.059
	Subspecies	-1.54 ± 0.572	-2.70	0.007
	NeS	-0.06 ± 0.024	-2.55	0.011
	L	0.09 ± 0.033	2.58	0.010
	Subspecies X NeS	0.05 ± 0.017	2.99	0.003
Time at less 1 meter	(intercept)	5.53 ± 0.746	7.42	<0.001
	Subspecies	0.08 ± 0.472	0.17	0.865
	NiS	0.004 ± 0.001	4.74	<0.001
	NiN	-0.04 ± 0.008	-4.27	<0.001
	NeN	-0.02 ± 0.004	-5.68	<0.001
	ReN	-0.02 ± 0.009	-2.23	0.026
	Subspecies X NiS	-0.002 ± 0.001	-4.15	<0.001
	Subspecies X NiN	0.02 ± 0.005	3.39	0.001
	Subspecies X NeN	0.01 ± 0.003	3.72	<0.001
Correct responses	(intercept)	3.21 ± 0.123	26.09	<0.001
	Subspecies	0.34 ± 0.076	4.48	<0.001
	NeS	-0.01 ± 0.001	-4.42	<0.001
	ReS	0.95 ± 0.341	2.78	0.005
	L	-0.04 ± 0.022	-1.95	0.052
	Subspecies X ReS	-0.94 ± 0.34	-2.75	0.006
	Subspecies X L	0.03 ± 0.013	2.04	0.042
Tail wagging	(intercept)	-2.45 ± 0.787	-3.12	0.002
	Subspecies	3.33 ± 0.49	6.80	<0.001
	NiS	0.02 ± 0.005	3.57	<0.001
	NeS	-0.05 ± 0.01	-4.66	<0.001
	ReS	1.57 ± 0.279	5.64	<0.001
	NeN	0.07 ± 0.02	3.43	0.001
	ReN	-0.56 ± 0.137	-4.09	<0.001
	L	0.09 ± 0.034	2.76	0.006
	Subspecies X NiS	-0.01 ± 0.003	-2.92	0.004
	Subspecies X NeS	0.03 ± 0.005	5.15	<0.001
	Subspecies X ReS	-1.62 ± 0.25	-6.48	<0.001
	Subspecies X NeN	-0.04 ± 0.01	-3.97	<0.001
	Subspecies X L	-0.04 ± 0.017	-2.54	0.011

*Explanatory variables without effect on the response variables were removed from the models during the model-selection process.
**Wolves or dogs; wolves is the reference group.

Table S4. Results of Dunn and Tukey *post hoc* tests comparing the acoustic parameters (minimum frequency, maximum frequency, delta time, peak frequency, number of speeches, average power) of nice, neutral and reprehensive speeches emitted by trainers in Positive Reinforcement Training sessions with dogs (*Canis familiaris*) and wolves (*Canis lupus*).

Post hoc	Bioacoustics variables		Values
Dunn	***Minimum frequency***	Neutral x Reprehensive	Mean difference = 4.98<br>p = 0.88
		Nice x Neutral *	Mean difference = 77.34<br>p < 0.001
		Reprehensive x Nice *	Mean difference = 82.32<br>p = 0.03
Dunn	***Maximum frequency***	Neutral x Reprehensive *	Mean difference = - 151.89<br>p < 0.001
		Nice x Neutral *	Mean difference = 78.04<br>p < 0.001
		Reprehensive x Nice *	Mean difference = - 73.85<br>p = 0.03
Tukey	***Average power***	Neutral x Reprehensive *	Mean difference = - 6.45<br>p = 0.00
		Nice x Neutral	Mean difference = - 0.54<br>p = 0.45
		Reprehensive x Nice *	Mean difference = - 6.70<br>p = 0.00
Dunn	***Delta time***	Neutral x Reprehensive *	Mean difference = -210.08<br>p < 0.001
		Nice x Neutral *	Mean difference = 95.47<br>p < 0.001
		Reprehensive x Nice *	Mean difference = -114.61<br>p < 0.001
Dunn	***Peak Frequency***	Neutral x Reprehensive *	Mean difference = -98.78<br>p = 0.01
		Nice x Neutral *	Mean difference = 112.47<br>p < 0.001
		Reprehensive x Nice	Mean difference = 13.70<br>p = 0.69
Dunn	***Number of Speeches***	Neutral x Reprehensive *	Mean difference = 235.89<br>p < 0.001
		Nice x Neutral *	Mean difference = 93.22<br>p < 0.001
		Reprehensive x Nice *	Mean difference = 329.11<br>p < 0.001

* Statistically significant results

Table S5. Final Generalized Linear Mixed Models* run for dogs (*Canis familiaris*) and wolves (*Canis lupus*), considering the effects of the explanatory variables (subspecies; minimum frequency – MinF; maximum frequency – MaxF; peak frequency – PF; average power – AP; delta time – DT and number of speeches – NS) on the response variables (visual orientation to trainer; exploring; jumping; time at less than 1 meter; correct responses and tail wagging).

Response variables	Explanatory variables	Estimate ± sd	z-value	*p*
Visual orientation to trainer	(intercept)	7.29 ± 0.79	9.25	<0.001
	Subspecies**	-0.81 ± 0.50	-1.63	0.104
	MinF	-0.006 ± 0.001	-5.93	<0.001
	AP	-0.03 ± 0.004	-8.02	<0.001
	DT	-0.45 ± 0.13	-3.52	<0.001
	PF	0.004 ± 0.001	4.47	<0.001
	Subspecies X MinF	0.003 ± 0.001	5.51	<0.001
	Subspecies X AP	0.015 ± 0.002	6.52	<0.001
	Subspecies X DT	0.24 ± 0.08	3.14	0.002
	Subspecies X PF	-0.002 ± 0.001	-4.13	<0.001
Exploring	(intercept)	6.97 ± 0.79	8.77	<0.001
	Subspecies	-3.52 ± 0.50	-6.98	<0.001
	MinF	0.012 ± 0.001	8.34	<0.001
	MaxF	0.005 ± 0.001	5.45	<0.001
	PF	-0.015 ± 0.002	-8.27	<0.001
Jumping	(intercept)	5.70 ± 3.95	1.44	0.15
	Subspecies	-4.04 ± 2.84	-1.42	0.154
	MinF	-0.012 ± 0.004	-2.95	0.003
	MaxF	-0.038 ± 0.008	-4.64	<0.001
	AP	0.24 ± 0.07	3.45	0.001
	DT	-7.12 ± 2.17	-3.29	0.001
	Subspecies X MaxF	0.024 ± 0.004	5.93	<0.001
	Subspecies X AP	-0.14 ± 0.05	-3.08	0.002
	Subspecies X DT	5.13 ± 1.40	3.67	<0.001
Time at less 1 meter	(intercept)	6.21 ± 0.77	8.09	<0.001
	Subspecies	-0.17 ± 0.48	-0.35	0.73
	MinF	-0.004 ± 0.001	-4.01	<0.001
	MaxF	-0.004 ± 0.001	-4.64	<0.001
	AP	-0.004 ± 0.002	-2.34	0.019
	DT	-0.31 ± 0.12	-2.56	0.011
	PF	0.006 ± 0.001	4.94	<0.001
	Subspecies X MinF	0.002 ± 0.001	3.58	<0.001
	Subspecies X MaxF	0.002 ± 0.0003	5.34	<0.001
	Subspecies X DT	0.20 ± 0.07	2.7	0.007
	Subspecies X PF	-0.003 ± 0.001	-4.94	<0.001
Correct responses	(intercept)	2.45 ± 0.51	4.79	<0.001
	Subspecies	0.71 ± 0.28	2.55	0.011
	MinF	-0.012 ± 0.003	-4.82	<0.001
	DT	-2.09 ± 0.43	-4.88	<0.001
	PF	0.012 ± 0.002	4.92	<0.001
	NS	0.005 ± 0.002	2.41	0.016
	Subspecies X MinF	0.008 ± 0.001	5.18	<0.001
	Subspecies X DT	0.96 ± 0.24	4.01	<0.001
	Subspecies X PF	-0.006 ± 0.001	-4.73	<0.001
	Subspecies X NS	-0.003 ± 0.001	-2.44	0.015
Tail wagging	(intercept)	4.65 ± 1.66	2.8	0.005
	Subspecies	-0.44 ± 0.90	-0.48	0.629

MinF	0.003 ± 0.001	5.01	<0.001
MaxF	0.056 ± 0.007	7.76	<0.001
AP	-0.23 ± 0.03	-8.45	<0.001
DT	6.28 ± 0.82	7.67	<0.001
PF	-0.063 ± 0.007	-9.28	<0.001
Subspecies X MaxF	-0.026 ± 0.004	-7.28	<0.001
Subspecies X AP	0.11 ± 0.01	8.07	<0.001
Subspecies X DT	-2.93 ± 0.41	-7.07	<0.001
Subspecies X PF	0.030 ± 0.003	8.68	<0.001

*Explanatory variables without effect on the response variables were removed from the models during the model-selection process.

**Wolves or dogs; wolves is the reference group.

Figure S2. Characterization of voice of the five trainers involved in Positive Reinforcement Training sessions with dogs (*Canis familiaris*) and wolves (*Canis lupus*). Left to right, bottom margin: trainers 1, 2, 3, 4 and 5. Acoustic parameters of trainers' voices: minimum frequency, maximum frequency, peak frequency, average power, delta time and number of speeches. Number of nice, neutral, and reprehensive speeches: 10.391, 7.500, 47 respectively.

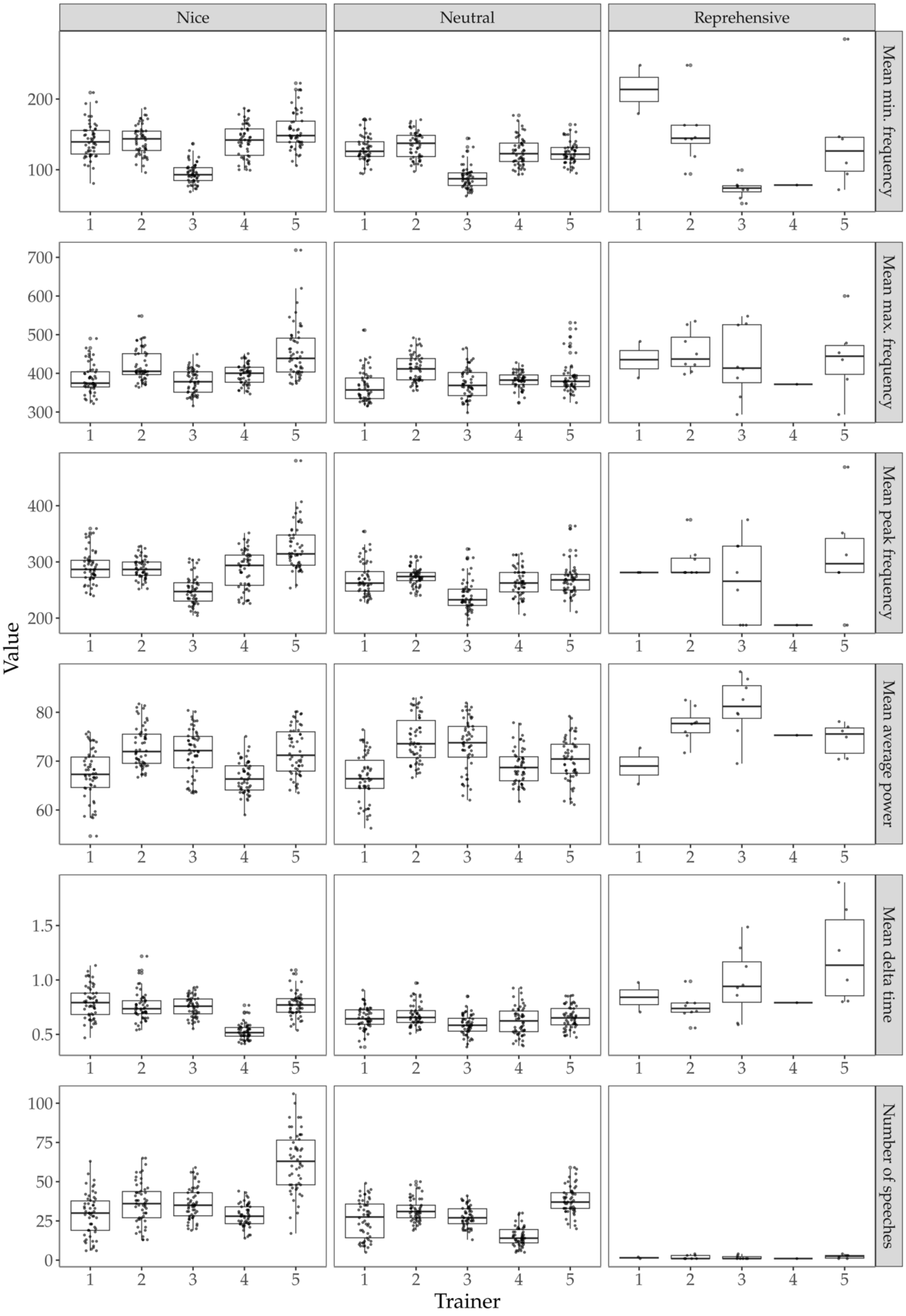

Supplement: Supplementary file 1 [file animals-13-01071-s001.zip › animals-2177895-supplementary.pdf]
